# Association between obesity and permanent teeth eruption in a sample of primary school children from Tanta City

**DOI:** 10.1186/s12903-025-06547-5

**Published:** 2025-07-18

**Authors:** Eman M. Esmael, Amina M. El-Hosary, Shaimaa S. EL-Desouky

**Affiliations:** Pediatric Dentistry, Oral Health and Preventive Dentistry Department, Faculty of Dentistry, Tanta University, Tanta, Egypt

**Keywords:** Dental eruption, Egyptian children, Obesity, Body mass index

## Abstract

**Introduction:**

Obesity is a major health threat of modern civilization with substantially increased prevalence which affects children’s health, growth, and development. Also, tooth eruption is certainly linked to the somatic development of the person.

**Aim:**

This study aimed to assess the association between the eruption of permanent teeth and obesity among a sample of primary school children from Tanta City.

**Methods:**

A case-control study was carried out on 2520 healthy students aged 6–12 years selected from 10 primary schools in Tanta City (equally included from public and private schools). The selected students were divided into equal sub-groups according to age, gender, and obesity status using a stratified random sampling technique. Obesity was assessed using BMI method while tooth eruption was recorded when any part of the crown was visible through the oral mucosa for twenty-eight permanent teeth. Statistical analysis was done for the collected data.

**Results:**

All permanent teeth in the maxillary and mandibular arches erupted earlier in private school children, except for the lower right second molar showed earlier eruption in public school children with no significant difference. The mean eruption age of all permanent teeth differed significantly between public and private institutions except for upper and lower right and left second molars, lower right first premolar, and lower right and left central incisors.

**Conclusion:**

This population-specific research revealed a highly significant positive association between the total number of permanent teeth erupted and BMI among school children aged 6–12 years from Tanta City.

## Introduction

Obesity is a major health issue that is impacting more than half a billion people globally and resulting in an array of health problems [1]. The World Health Organization (WHO) described the marked increase in body weight as a ‘global epidemic disease’. Obesity can be defined as a condition in which energy intake becomes higher than the required energy, leading to body fat deposition [2].

Overweight and obesity are multifactorial diseases with the most prevalent risk factors being genetic factors. Increased obesity is associated with many causes like excessive beverage intake, overeating junk foods and sugary meals with a lack of exercise, and spending hours playing video games and watching TV. Parenting factors, lower socioeconomic groups, and psychological problems also result in overweight and obesity [3]. Body Mass Index (BMI) is the best indirect, noninvasive, and precise method to assess body fat. BMI is the ratio of body weight in kilograms (kg) to height in meters squared (m^2^), which is used to classify obesity and overweight [4].

Tooth eruption is “the movement of the tooth from its developmental site in the alveolar bone to its functional position in the oral cavity which is necessary for the survival of diverse species“ [5]. Permanent tooth eruption is a complex process that takes place across a wide age range and is impacted by several general, demographic, and local factors. The general factors include genetics, diet, premature birth, economic status, body height and weight, hormonal variables, and a variety of systemic disorders. Demographic factors also influence tooth eruption in children and adolescents such as age, race, and gender [6]. The important local factors influencing tooth eruption are supernumerary teeth, crowding, arch length deficiency, odontogenic cysts, and tumors, dentigerous cysts, enamel pearls, gingival hyperplasia, premature loss of a primary tooth, ankylosis, thumb sucking, tongue thrusting, eruption cysts, eruption sequestra and fibrous developmental malformations [7].

Faster dental development has been linked with higher adrenal androgen release with a positive association between body height and weight and tooth emergence has been found in previous research [8, 9]. Taller and heavier youngsters have early eruptions, whereas stunting (delayed linear growth) is correlated with delaying tooth eruption. So, the eruption of teeth is significantly influenced by the child’s general development [10]. A 4-year longitudinal study carried out by Sánchez-pérez et al., [11] found an association between body mass index (BMI) and tooth eruption in a cohort of Mexican schoolchildren as children with higher BMI had more erupted teeth compared to other children. Moreover, A Spanish study by Traver-Ferrando and Barcia-González [12]., on Valencian children obesity and dental eruption showed a positive correlation in which obese children mature earlier and teeth tend to erupt on average 1.2 to 1.5 years earlier as compared to children with normal body mass index. A cross-sectional study conducted by Lock et al., [13] reported a significant association between overweight/obesity at age 12 and early teeth eruption at ages 12 and 14–15 in schoolchildren in southern Brazil. Another cross-sectional study by Padmanabhan et al., [14] found a significant correlation between BMI and the age at which permanent teeth emerge in school-aged children aged 6 to 14 who visited a dental hospital in UAE.

As it is proposed that obesity is linked to early maturation, hence it is likely to impact tooth eruption [15]. To our knowledge, few studies have been done on the relation between obesity and permanent teeth eruption among Egyptian children, so this study was directed to investigate the relation between the eruption of permanent teeth and obesity in Egyptian children through a sample of primary school children from Tanta City. The null hypothesis (H_0_) assumed that there was no significant correlation between the eruption of permanent teeth and obesity in primary school children from Tanta City.

## Materials and methods

### Study setting and ethical consideration

The study was carried out as a case-control study on a sample of primary school students (public and private) in Tanta City from October 2022 to January 2023. This study was approved by the Research Ethics Committee at Tanta University’s Faculty of Dentistry, code (#PED-08-22-4), in accordance with the 1964 Helsinki Declaration and its amendments. Also, agreement was obtained from Education affairs and school authorities. After mailing letters explaining the purpose of the study, parents signed written informed consent to examine their children.

### Sample size calculation

The sample size was computed based on a previous study by Šindelářová et al., [16] using the G-power version 3.1.9 computer program. The study’s power had been set at 95.02%, and the alpha level was 0.05. The estimated minimum sample size(n) was 2072 children, then it was increased to 2520 children to compensate for the potential failure and improve the validity of results.

### Sample description

**Fig. 1:**
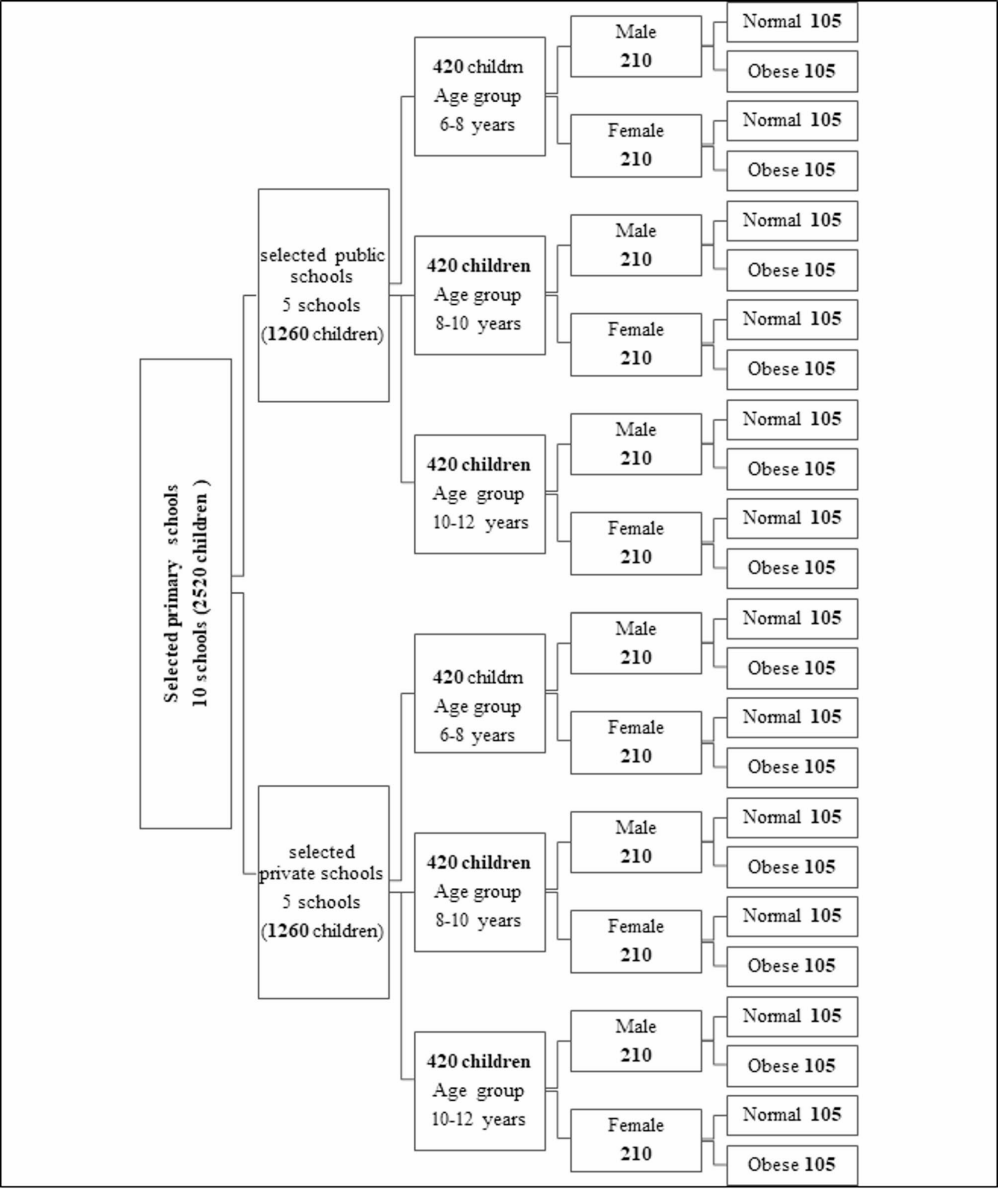
A stratified random sampling technique was used in this study

A total of 2520 healthy Egyptian students with an age range of 6–12 years were selected at random from five public and five private schools in Tanta City in a stratified random manner as shown in Fig. 1. Children were excluded from the study if they were non-Egyptian nationals or had systemic conditions, such as endocrine disorders, that could affect growth and dental development. Exclusion criteria also included cleft lip and palate, congenital dental anomalies (e.g., supernumerary, missing, or malformed teeth), and any prior medical treatments (e.g., steroids, chemotherapy, or radiotherapy) [17]. Additionally, children with a history of orthodontic treatment, permanent tooth extraction, or traumatic loss of permanent teeth (as determined by clinical examination and reported dental history) were precluded. Underweight or malnourished children (BMI below the 5th percentile) were also not included.

Selected students were divided into two main equal groups according to school type (1260 students for each), consequently, each of the two groups was assigned into three sub-groups according to their age:

- **Subgroup-A**: from 6 to less than 8 years old, including 420 students.
- **Subgroup-B**: from 8 to less than 10 years old, including 420 students.
- **Subgroup-C**: from 10 to less than 12 years old, including 420 students.

After that, each subgroup was divided into male and female groups (210 students) according to gender, then into overweight/obese and normal-weight children (105 students).

### Data collection

A pre-survey calibration was performed on 25 school students to assess intra-examiner reliability by examining the same students twice in weekly intervals. Kappa values for all observations were greater than 0.91, indicating high intra-examiner reliability.

On the first visit to the school, students were asked to contribute to the study, sampled, and given consent letters to their parents containing basic details about the study’s purpose and the children’s upcoming physical examination. On the next visit, consent letters were gathered from the students then, obesity and tooth eruption were assessed only for the allowed students to share in the study. The child’s chronological age was calculated by subtracting his or her date of birth from the day of examination; the child’s birth date was obtained from school records. The collected data for each student was recorded in a predesigned examination chart.

### Obesity assessment

Obesity (body weight & height) was evaluated by a skilled and standardized examiner (first author). Body height was recorded to the nearest 0.001 m with a length-measuring device (Mejozar, Granzia, Italy) in a straight position, without shoes or caps, with the back, shoulder, hips, and legs and heels completely tangent to the caliber and facing forward. The body weight was recorded in kilograms (kg) to the second decimal number using a self-zeroing digital scale (Prestige, Granzia, Italy). The children stood in the center of the scale, dressed minimally with no shoes, hats, or other accessories, and their weight was recorded. The formula for calculating the body mass index (BMI) was weight in kilograms divided by height in meters squared (kg/m^2^) [18] which was plotted on BMI-for-growth charts of the Centers for Disease Control and Prevention (CDC) that are gender specific. Once plotted, the percentile can be determined. The American Obesity Association (AOA) uses the 5th percentile to less than the 85th percentile for normal weight, the 85th to less than the 95th percentile for overweight which corresponds to a BMI of 25 kg/m^2,^ while equal to or greater than the 95th percentile refers to obesity which corresponds to a BMI of 30 kg/m^2^ [14, 19].

The BMI scores were dichotomized into two categories (‘normal-weight’ or ‘overweight/obese’) according to the World Health Organization [10, 20] as follows: Overweight/obese children: BMI z-score > 85th percentile;

Normal-weight children: BMI z-score ≥ 5 and ≤ 85th percentile.

### Teeth eruption assessment

The selected children were examined on a portable chair in their schools after brushing their teeth then, the eruption of 28 permanent teeth (2 incisors, 1 canine, 2 premolars, and 2 molars in each quadrant of the mouth) was examined clinically using an intraoral disposable mirror with a light source by a trained, calibrated examiner (first author). The clinical eruption was recorded when there was a visible breakthrough of any part of the tooth crown through the gingival mucosa [21].

### Statistical analysis

Data was collected and tabulated in Excel sheets and then analyzed using Statistical Program for Social Science (SPSS) version 22.0. Kolmogorov-Smirnov test of normality was done, and the data was normally distributed. Quantitative data were expressed as mean and standard deviation (SD). Qualitative data were expressed as frequency and percentage. The Chi-square test was used to compare categorical variables. For comparisons between continuous variables, independent-sample t-tests and one-way ANOVA were applied as appropriate. To evaluate the relationship between body mass index (BMI) and the number of erupted permanent teeth, Pearson’s correlation coefficient was used. Also, a multiple linear regression analysis was conducted to examine the influence of school type, age group, gender, and BMI status on the mean number of erupted permanent teeth. These regression models help identify which variables are significantly associated with dental eruption and determine the extent to which each variable contributes to explaining the variation in the outcome. A p-value less than 0.05 was considered statistically significant for all analyses.

## Results

A comparison between the mean number of erupted permanent teeth for overweight/obese and normal-weight children in public and private schools of different age groups was presented in Table 1. There were highly significant statistical differences between public and private normal-weight children in the age groups of 6–8 and 8–10 years, while a non-significant difference in the age group of 10–12 years was noted. Also, there was a highly significant difference between public and private overweight/obese children in the age groups of 8–10 and 10–12 years while a non-significant difference in the age group 6–8 years was observed (Table 1).

**Table 1:** Comparison of the mean number of erupted permanent teeth in different age groups by BMI status and school type

Age group	BMI	School type	Range	Mean ± SD	t-test	*p*-value
6–8 years	Normal-weight	Public	0–11	4.70 ± 1.98	4.332	0.001*
		Private	0–13	5.65 ± 2.50		
	Overweight/Obese	Public	2–12	6.50 ± 1.90	0.431	0.667
		Private	0–13	6.41 ± 2.58		
8–10 years	Normal-weight	Public	2–15	8.34 ± 2.23	3.145	0.002*
		Private	6–20	9.02 ± 2.21		
	Overweight/Obese	Public	3– 18	9.80 ± 2.75	8.757	0.001*
		Private	6–27	12.70 ± 3.94		
10–12 years	Normal-weight	Public	8–28	14.80 ± 4.07	1.491	0.137
		Private	8–27	15.32 ± 3.06		
	Overweight/Obese	Public	6 − 28	16.98 ± 5.15	3.157	0.002*
		Private	11–28	18.46 ± 4.47		

SD: standard deviation * Statistically significant difference (*p* < 0.05)

As shown in Table 2, in public schools, there were highly statistically significant differences between normal and overweight/obese children in age groups 6–8 and 10–12 years in both genders (*p* < 0.001) except female children in the age group 8–10 years (*p* = 0.208). while in private schools, there were highly statistically significant differences between normal and overweight/obese children in age groups 8–10 and 10–12 years in both genders (*p* < 0.001) except female children in age group 6–8 years (*p* = 0.698).

**Table 2:** Comparison of the mean number of erupted permanent teeth in public and private school children by age category, gender, and BMI status

School Type	Age Group	Gender	BMI Status	Range	Mean ± SD	t-test	*p*-value
Public	6–8 yrs	Male	Normal-weight	0–11	5.43 ± 1.89	3.223	0.001*
			Overweight/Obese	2–10	6.27 ± 1.88		
		Female	Normal-weight	0–8	3.97 ± 1.80	10.861	0.001*
			Overweight/Obese	3–12	6.74 ± 1.90		
	8–10 yrs	Male	Normal-weight	2–13	7.86 ± 2.23	7.537	0.001*
			Overweight/Obese	3–18	10.59 ± 2.97		
		Female	Normal-weight	4–15	8.83 ± 2.14	0.761	0.208
			Overweight/Obese	5–15	9.06 ± 2.21		
	10–12 yrs	Male	Normal-weight	8–23	13.29 ± 2.73	3.230	0.001*
			Overweight/Obese	6–28	15.00 ± 4.70		
		Female	Normal-weight	10–28	16.31 ± 4.60	4.053	0.001*
			Overweight/Obese	12–28	18.95 ± 4.83		
Private	6–8 yrs	Male	Normal-weight	0–13	4.72 ± 2.73	3.455	0.001*
			Overweight/Obese	0–13	6.14 ± 3.20		
		Female	Normal-weight	3–12	6.58 ± 1.83	0.389	0.698
			Overweight/Obese	4–11	6.68 ± 1.72		
	8–10 yrs	Male	Normal-weight	6–20	9.20 ± 2.72	6.810	0.001*
			Overweight/Obese	6–27	12.48 ± 4.11		
		Female	Normal-weight	6–15	8.80 ± 1.57	10.378	0.001*
			Overweight/Obese	7–24	12.92 ± 3.76		
	10–12 yrs	Male	Normal-weight	8–21	13.67 ± 2.52	6.201	0.001*
			Overweight/Obese	12–28	16.74 ± 4.41		
		Female	Normal-weight	11–27	16.98 ± 2.64	7.047	0.001*
			Overweight/Obese	11–28	20.18 ± 3.83		

SD: standard deviation; * statistically significant difference (*p* < 0.05)

Overweight/obese children exhibited earlier eruption of all maxillary permanent teeth compared to their normal-weight peers. The difference in mean age of eruption was highly significant (*p* < 0.001) for all upper teeth, except the right and left second molars, as shown in Table 3. Similarly, all mandibular permanent teeth erupted earlier in overweight/obese children, with statistically significant differences compared to normal-weight children, as presented in Table 4.

**Table 3:** Comparison of the mean age of eruption of permanent maxillary teeth in different obesity statuses

Teeth	BMI	*N*	Range	Mean ± SD	t-test	*p*-value
**UR 11**	Normal-weight	841	6.09–12	9.82 ± 1.62	6.489	0.001*
	Overweight/Obese	975	6.03–11.99	9.35 ± 1.40		
**UR 12**	Normal-weight	425	6.37–12	10.79 ± 1.03	9.243	0.001*
	Overweight/Obese	558	6.1–11.99	10.16 ± 1.08		
**UR 13**	Normal-weight	82	7.08–11.97	11.06 ± 1.02	5.679	0.001*
	Overweight/Obese	141	7.16–11.97	10.30 ± 0.93		
**UR 14**	Normal-weight	278	6.31–11.98	10.65 ± 0.97	7.779	0.001*
	Overweight/Obese	488	6.76–11.99	10.01 ± 1.16		
**UR 15**	Normal-weight	73	8.95–11.98	10.90 ± 0.76	4.741	0.001*
	Overweight/Obese	171	7.92–11.97	10.29 ± 0.98		
**UR 16**	Normal-weight	1101	6.03–12	9.35 ± 1.72	4.358	0.001*
	Overweight/Obese	1174	6.03–11.99	9.06 ± 1.45		
**UR 17**	Normal-weight	15	6.63–11.97	10.78 ± 1.74	0.986	0.327
	Overweight/Obese	55	8.03–11.83	10.46 ± 0.88		
**UL 21**	Normal-weight	830	6.05–12	9.89 ± 1.54	7.741	0.001*
	Overweight/Obese	979	6.1–11.99	9.36 ± 1.37		
**UL 22**	Normal-weight	411	6.08–12	10.63 ± 1.28	5.518	0.001*
	Overweight/Obese	547	6.19–11.99	10.22 ± 1.02		
**UL 23**	Normal-weight	61	9.25–11.98	11.12 ± 0.56	6.631	0.001*
	Overweight/Obese	145	8.03–11.97	10.28 ± 0.92		
**UL 24**	Normal-weight	241	7.72–12	10.77 ± 0.86	8.329	0.001*
	Overweight/Obese	444	6.61–11.99	10.07 ± 1.14		
**UL 25**	Normal-weight	62	8.59–11.98	11.11 ± 0.61	5.987	0.001*
	Overweight/Obese	172	7.64–11.97	10.37 ± 0.90		
**UL 26**	Normal-weight	1113	6.03–12	9.35 ± 1.70	4.418	0.001*
	Overweight/Obese	1185	6.03–11.99	9.06 ± 1.44		
**UL 27**	Normal-weight	13	9.25–11.97	10.85 ± 0.86	1.669	0.100
	Overweight/Obese	58	8.03–11.63	10.41 ± 0.86		

SD: standard deviation * Statistically significant difference (*p* < 0.05)

**Table 4:** A comparison of the mean age of eruption of permanent mandibular teeth in different obesity statuses

Teeth	BMI	*N*	Range	Mean ± S. D	t-test	*p*-value	
**LL 31**	Normal-weight	1021	6.05–12	9.57 ± 1.63	7.042		0.001*
	Overweight/Obese	1167	6.03–11.99	9.11 ± 1.42			
**LL 32**	Normal-weight	549	6.37–12	10.58 ± 1.20	11.359		0.001*
	Overweight/Obese	729	6.1–11.99	9.78 ± 1.28			
**LL 33**	Normal-weight	167	8.18–11.98	10.85 ± 0.66	5.728		0.001*
	Overweight/Obese	232	8.03–11.93	10.38 ± 0.90			
**LL 34**	Normal-weight	207	6.17–11.98	10.71 ± 1.12	6.718		0.001*
	Overweight/Obese	320	6.61–11.97	10.06 ± 1.06			
**LL 35**	Normal-weight	58	7.47–11.98	10.91 ± 0.77	2.781		0.006*
	Overweight/Obese	161	8.03–11.93	10.57 ± 0.81			
**LL 36**	Normal-weight	1217	6.03–12	9.15 ± 1.77	2.461		0.014*
	Overweight/Obese	1244	6.03–11.99	8.99 ± 1.45			
**LL 37**	Normal-weight	21	9.25–11.97	10.95 ± 0.67	2.553		0.012*
	Overweight/Obese	79	8.03–11.75	10.46 ± 0.81			
**LR 41**	Normal-weight	1134	6.03–12	9.25 ± 1.75	20,412		0.016*
	Overweight/Obese	1151	6.03–11.99	9.13 ± 1.41			
**LR 42**	Normal-weight	622	6.23–12	10.40 ± 1.30	10.239		0.001*
	Overweight/Obese	788	6.03–11.99	9.68 ± 1.32			
**LR 43**	Normal-weight	236	8.18–11.99	10.98 ± 0.65	9.008		0.001*
	Overweight/Obese	282	8.03–11.93	10.40 ± 0.79			
**LR 44**	Normal-weight	109	7.42–11.98	10.73 ± 0.89	4.672		0.001*
	Overweight/Obese	245	6.59–11.97	10.21 ± 1.00			
**LR 45**	Normal-weight	36	9.25–11.98	10.97 ± 0.68	3.668		0.001*
	Overweight/Obese	126	8.03–11.59	10.46 ± 0.75			
**LR 46**	Normal-weight	1237	6.03–12	9.13 ± 1.77	2.001		0.045*
	Overweight/Obese	1235	6.03–11.99	9.00 ± 1.44			
**LR 47**	Normal-weight	6	10.2–11.97	11.26 ± 0.69	2.872		0.006*
	Overweight/Obese	67	8.75–11.64	10.64 ± 0.49			

SD: standard deviation * Statistically significant difference (*p* < 0.05)

As shown in Table 5, in the maxillary arch, all permanent teeth emerged earlier in females than in males except for the right lateral incisor, left canines, and right second molar. In addition, there was a significant difference between males and females in the mean age of eruption of the upper right and left central incisors, upper left lateral, and upper left second molar (Table 5).

**Table 5:** Comparison of the mean age of eruption of maxillary permanent teeth between male and female children

Teeth	Sex	*N*	Range	Mean ± SD	t-test	*p*-value
**UR 11**	Male	878	6.03-12	9.68 ± 1.53	3.076	0.002*
	Female	938	6.07–11.99	9.46 ± 1.51		
**UR 12**	Male	515	6.1–12	10.38 ± 1.22	1.418	0.155
	Female	468	7.08–11.99	10.48 ± 0.95		
**UR 13**	Male	56	7.16–11.97	10.42 ± 1.33	1.318	0.188
	Female	167	7.08–11.97	10.63 ± 0.91		
**UR 14**	Male	341	7.39–11.99	10.35 ± 1.19	2.301	0.022*
	Female	425	6.31–11.98	10.16 ± 1.09		
**UR 15**	Male	87	8.01–11.98	10.54 ± 1.08	0.162	0.876
	Female	157	7.92–11.97	10.43 ± 0.88		
**UR 16**	Male	1132	6.03-12	9.26 ± 1.62	1.786	0.073
	Female	1143	6.03–11.99	9.14 ± 1.57		
**UR 17**	Male	23	8.03–11.75	10.59 ± 1.18	0.352	0.727
	Female	47	6.63–11.97	10.49 ± 1.09		
**UL 21**	Male	864	6.1–12	9.72 ± 1.50	3.172	0.002*
	Female	945	6.05–11.99	9.50 ± 1.45		
**UL 22**	Male	504	6.19-12	10.47 ± 1.13	2.011	0.045*
	Female	454	6.08–11.98	10.32 ± 1.18		
**UL 23**	Male	61	8.03–11.97	10.55 ± 1.06	0.213	0.832
	Female	145	8.25–11.98	10.52 ± 0.86		
**UL 24**	Male	290	6.61-12	10.37 ± 1.18	1.172	0.241
	Female	395	6.76–11.98	10.27 ± 1.04		
**UL 25**	Male	87	8.03–11.98	10.71 ± 0.94	1.912	0.057
	Female	147	7.64–11.98	10.48 ± 0.86		
**UL 26**	Male	1148	6.03-12	9.25 ± 1.61	1.368	0.171
	Female	1150	6.03–11.99	9.16 ± 1.54		
**UL 27**	Male	31	8.03–11.63	10.23 ± 1.15	2.259	0.027*
	Female	40	10.01–11.97	10.69 ± 0.51		

SD: standard deviation* Statistically significant difference (*p* < 0.05)

In the mandibular arch, females showed earlier eruptions of lower right central incisors, right and left first premolars, and right and left first molars as illustrated in Table 6. Furthermore, a highly substantial statistical difference was found between both genders with the mean age of eruption of the lower right and left canines and lower right central incisor (Table 6).

**Table 6:** Comparison of the mean age of eruption of mandibular permanent teeth between male and female children

Teeth	Sex	*N*	Range	Mean ± SD	t-test	*p*-value
**LL 31**	Male	1096	6.03-12	9.35 ± 1.59	0.762	0. 449
	Female	1083	6.05–11.99	9.30 ± 1.49		
**LL 32**	Male	690	6.1–12	10.07 ± 1.41	1.502	0.133
	Female	588	6.42–11.99	10.18 ± 1.17		
**LL 33**	Male	87	8.03–11.93	10.33 ± 1.12	3.178	0.002*
	Female	312	8.11–11.98	10.65 ± 0.73		
**LL 34**	Male	213	6.61–11.98	10.40 ± 1.13	1.402	0.163
	Female	314	6.17–11.98	10.26 ± 1.13		
**LL 35**	Male	63	8.03–11.79	10.50 ± 1.04	1.829	0.069
	Female	156	7.47–11.98	10.72 ± 0.69		
**LL 36**	Male	1231	6.03-12	9.13 ± 1.62	2.001	0.046*
	Female	1230	6.03–11.99	9.00 ± 1.60		
**LL 37**	Male	39	8.03–11.79	10.35 ± 1.12	0.231	0.028*
	Female	61	10.01–11.97	10.71 ± 0.47		
**LR 41**	Male	1113	6.03-12	9.31 ± 1.61	2.859	0.004*
	Female	1172	6.03–11.99	9.12 ± 1.57		
**LR 42**	Male	724	6.03-12	9.99 ± 1.47	0.279	0.782
	Female	686	6.23–11.99	10.01 ± 1.23		
**LR 43**	Male	83	8.03–11.89	10.24 ± 1.08	5.618	0.001*
	Female	435	8.18–11.99	10.75 ± 0.68		
**LR 44**	Male	142	6.59–11.98	10.43 ± 1.08	0.841	0.404
	Female	212	7.51–11.98	10.34 ± 0.93		
**LR 45**	Male	53	8.03–11.69	10.46 ± 0.97	1.332	0.185
	Female	109	8.26–11.98	10.63 ± 0.64		
**LR 46**	Male	1236	6.03-12	9.12 ± 1.63	0.241	0.810
	Female	1236	6.03–11.99	9.01 ± 1.60		
**LR 47**	Male	22	8.75–11.52	10.62 ± 0.62	0.809	0.420
	Female	51	10.01–11.97	10.73 ± 0.49		

SD: standard deviation * Statistically significant difference (*p* < 0.05)

A statistically significant positive correlation was found between BMI and the total number of erupted permanent teeth across all age groups (*p* < 0.001) (Table 7). overweight/obese children consistently had a greater mean number of erupted teeth compared to their normal-weight counterparts at each age.

**Table 7:** Correlation between the total number of permanent teeth and BMI in each age group

BMI	Total No. permanent teeth	
	*R*	*P*-value
6–8 years	0.263	0.001*
8–10 years	0.376	0.001*
10–12 years	0.299	0.001*

Table 8 showed the results of a multiple linear regression analysis evaluating the effects of school type, age group, gender, and BMI status on the mean number of erupted permanent teeth. The regression model was statistically significant with an *R*^*2*^ value of 0.812, indicating that approximately 81.2% of the variance in the number of erupted teeth was explained by the predictors. The age group was found to be the strongest predictor of erupted teeth (*p* = 0.001), indicating a strong positive association between increasing age and the number of erupted teeth. Followed by BMI status (*p* = 0.001) also had a notable positive effect, with overweight/obese children exhibiting more erupted teeth than their normal-weight counterparts. Then, gender (*p* = 0.015) had a positive effect with females having significantly more erupted teeth compared to males. Finally, school type (*p* = 0.032) had a positive coefficient, suggesting that children in private schools may have more erupted teeth than in public schools. This suggested that children who were older, overweight/obese, female, and in private school had a higher number of erupted permanent teeth.

**Table 8:** Multiple linear regression analysis of factors associated with the number of permanent teeth erupted

Variables	Coefficients	Std. Error	t-value	*p*-value	95% CI Lower	95% CI Upper
Constant	-4.351	0.332	-13.086	0.001	-5.003	-3.699
Age	5.287	0.080	66.205	0.001	5.130	5.443
Gender	1.219	0.130	9.348	0.015	0.963	1.475
BMI	2.146	0.130	16.457	0.001	1.890	2.402
School type	1.067	0.130	8.180	0.032	0.811	1.322

R^2^ = 0.812 Adjusted R^2^= 0.808

## Discussion

Childhood obesity is a major health issue in the pediatric population. The impact of obesity on the eruption time of permanent dentition has great clinical importance for dental treatment planning in both orthodontic and pediatric dentistry [22]. Accelerated teeth eruption in children with obesity may increase the liability to dental caries because of the increased retention period of teeth in the oral cavity [23].

This study was directed to investigate the correlation between the eruption of permanent teeth with obesity in a sample of primary school children from Tanta City. The age group selected for this study was 6–12 years old since permanent teeth eruption has been an age-specific phenomenon starting from 6 years of age and ending by 12 years of age excluding the third molars. This is in line with Kutesa et al., [24] who studied permanent dentition in Ugandan children aged 5–14, Díaz-Orahulio et al., [25] who studied the tooth eruption sequence and nutritional health of children younger than 12 years old, and Raghavan et al., [26] who studied the relation between the eruption of the permanent teeth and body mass index in Chennai City in school children aged 7–17 years.

The eruption of permanent teeth in this study was assessed by clinical visual examination as the radiographical equipment was not available in schools; this agreed with Wong et al., [27], and Reis et al., [28]. While it disagreed with Hilgers et al., [29] who used panoramic X-ray, Nicholas et al., [30] who used periapical radiographs to assess dental development for children with increased BMI values, and Paz-Cortez et al., [21] used both visual examination and orthopantomography. In this study, child obesity was measured using BMI since BMI is mostly applied for the categorization of childhood obesity; this agreed with Nicholas et al., [30], Dimaisip-Nabuab et al., [31]. While this contrasted with Wong et al., [27] who determined children’s obesity using five anthropometric indices.

Regarding the mean number of erupted permanent teeth, the present study results revealed significant statistical differences between both groups with overweight/obese children having a higher number of erupted teeth. This is in line with Evangelista et al., [10] who concluded that the overweight/obesity group was associated with a significantly larger mean number of erupted permanent teeth (*p* < 0.001). Also, this agreed with Must et al., [32] who found that compared to non-obese, obese children had 1.44 more permanent teeth erupting throughout the mixed dentition phase.

Concerning the mean age of eruption, the current study findings reported statistically substantial differences between both groups for all permanent teeth except for maxillary second molars, with overweight/obese children having earlier teeth eruption than normal-weight children. This may be explained by the fact that a high body mass value can affect dental eruption as overall growth appears to be faster in obese children, particularly skeletal growth and the age at first menarche in girls [33]. Moreover, adipocytes secrete leptin hormone, which controls appetite and the body’s energy balance; in obese children, leptin directly contributes to skeletal growth and plays a role in regulating bone metabolism, pubertal development, and linear growth [10, 34]. A low-grade chronic inflammation linked to obesity may also disrupt signaling pathways that regulate tooth eruption, contributing to early permanent tooth emergence [35]. Furthermore, a diet characterized by nutrient-poor but energy-dense alternatives, and genetic variables all have an impact on tooth development and eruption time [14]. This agreed with Hilgers et al., [29] and Bagwedi et al., [36]. who clarified that obese children had a quicker dental eruption. Moreover, Sánchez-Pérez et al., [37] suggested that being overweight quickens the permanent teeth eruption significantly. Also, this is in line with Hedayati et al., [9] who reported that increased BMI showed early dental eruption. On the other hand, this is contrasted to Anu et al., [38] who revealed a negative association, indicating a delay in dental eruption in obese children as opposed to normal weight. Also, this was opposed to Khan [39]., who found that a non-significant association was observed between BMI and the mean age of eruption, except for lower lateral incisors. Furthermore, this disagreed with Raghavan et al., [26] who concluded that the " mean age of eruption increased significantly with increased BMI indicated delayed eruption among obese children.”

In relation to gender, the current study results revealed that female children had all maxillary permanent teeth erupted earlier than males except for the right lateral incisor, left canines, and right second molar. Also, in the mandibular arch, females showed earlier eruptions of right central incisors and left & right first premolars and first molars. This may be attributed to the earlier onset of maturation in females which affects the tooth eruption timing. This coincided with Raghavan et al., [26] who stated that girls demonstrated an earlier eruption in comparison to boys. Also, this is in line with Sapunarova et al., [40] who concluded a considerable correlation between children’s gender, and eruption in favor of females, and added that being female accelerates dental eruption. While this disagreed with Bagewadi et al., [36] who concluded " early eruption of the maxillary canine, mandibular second premolar, maxillary, and mandibular second molars among male children.” Additionally, this study’s results reported earlier eruption of mandibular teeth than maxillary teeth in both genders. This may be explained by the fact that the maxilla and mandible have different bone structures with the former being less dense and thinner, particularly in the anterior area, which causes the mandibular teeth to erupt earlier than their maxillary counterparts [41]. Based on the pace of tooth development, mandibular tooth crowns require less time to complete than maxillary tooth crowns also, the permanent mandibular incisor tooth buds are positioned lingually to the primary incisors, allowing them to emerge into the oral cavity before the primary incisors exfoliate [42]. This agreed with Gupta et al., [43] who observed that in both sexes, the mandibular teeth emerge before the maxillary teeth.

The present study revealed that all permanent teeth in the upper and lower arch erupted earlier in private school children, except for the lower right second molar; this agreed with Khan [39]., who concluded earlier tooth eruptions in private school children. This may be explained by children of low socio-economic status with malnutrition usually enrolling in public schools so, public school children usually have delay in eruption than the children grow up with a healthy diet [44].

This study’s results rejected the null hypothesis because there was a highly significant positive correlation between the total number of permanent teeth erupted and BMI for each age category in primary school children in Tanta City suggesting that increased body mass may be linked to accelerated dental development. This may be clarified by higher leptin levels in obesity that accelerate skeletal and dental growth, whereas pro-inflammatory adipokines further exaggerate these changes [45]. Moreover, in obese individuals, the expansion of adipose tissue can trigger hormonal alterations such as elevated levels of insulin-like growth factor-1 that contributes significantly to the acceleration of bone maturation [46]. Also, metabolic shifts including changes in mineral metabolism may contribute to an accelerated rate of tooth eruption [47]. This agreed with Hernandez et al., [48] who found a positive correlation for both genders and in all age categories in Spanish children. Also, this is in line with Gaur et al., [49] who reported a direct correlation between Mexican children’s weight in girls, and boys with 46%, and 64% of them respectively. On the other hand, this disagreed with Eid et al., [50] who reported a non-significant association between BMI and dental maturation in Brazilian children. Moreover, Kutesa et al., [24] revealed no correlation between BMI and permanent teeth eruption.

The study’s limitation was that it did not include data on the timing of obesity onset, which may influence the timing of permanent tooth eruption. Additionally, the absence of radiographic evaluation was a limitation, as it restricted the ability to assess underlying local factors that may affect individual eruption timing and sequence such as the presence of certain tooth anomalies (e.g., supernumerary teeth) also, the assessment of root development. Furthermore, the study focused solely on the eruption of permanent teeth, excluding primary dentition. Future research should incorporate radiographic evaluations and study models for more comprehensive assessments. Longitudinal and multicenter studies with larger sample sizes, covering various regions of Egypt, are also recommended to validate and expand upon these findings in both primary and permanent dentitions.

## Conclusion

The findings of this study demonstrate a significant association between childhood obesity and accelerated eruption of permanent teeth. Overweight/obese children exhibited a higher number of erupted teeth compared to their normal-weight peers across all age groups, with girls showing earlier eruption patterns than boys. Additionally, children from private schools generally experienced earlier tooth eruption than those from public schools. These results underscore the importance of incorporating early caries prevention strategies, particularly for overweight/obese children who may be at increased risk due to earlier tooth eruption. Dental professionals should consider these eruption trends when planning preventive and educational programs targeted at high-risk pediatric populations.

## Data Availability

The datasets used and/or analysed in this study are available from the corresponding author upon reasonable request.

## Declarations

Before the start of the study, an agreement was gained from the Research Ethics committee at the Faculty of Dentistry, Tanta University, code: (#PED-08-22-4), this complied with the Helsinki Declaration of 1964 and its subsequent amendments. Also, agreements were obtained from Education affairs, and school authorities. Informed written consent to examine children was signed from their parents and their children after mailing letters to clarify the aim of the present study.
